# Augmenting LTP-Like Plasticity in Human Motor Cortex by Spaced Paired Associative Stimulation

**DOI:** 10.1371/journal.pone.0131020

**Published:** 2015-06-25

**Authors:** Florian Müller-Dahlhaus, Caroline Lücke, Ming-Kuei Lu, Noritoshi Arai, Anna Fuhl, Eva Herrmann, Ulf Ziemann

**Affiliations:** 1 Department of Neurology, Goethe-University Frankfurt, Germany; 2 Department of Neurology and Stroke, and Hertie Institute for Clinical Brain Research, Eberhard-Karls-University Tübingen, Germany; 3 Department of Neurology, China Medical University Hospital, Taichung, Taiwan; 4 Institute of Biostatistics and Mathematical Modelling, Goethe-University Frankfurt, Germany; Hospital Nacional de Parapléjicos, SPAIN

## Abstract

Paired associative stimulation (PAS_LTP_) of the human primary motor cortex (M1) can induce LTP-*like* plasticity by increasing corticospinal excitability beyond the stimulation period. Previous studies showed that two consecutive PAS_LTP_ protocols interact by homeostatic metaplasticity, but animal experiments provided evidence that LTP can be augmented by repeated stimulation protocols spaced by ~30min. Here we tested in twelve healthy selected PAS_LTP_ responders the possibility that LTP-*like* plasticity can be augmented in the human M1 by systematically varying the interval between two consecutive PAS_LTP_ protocols. The first PAS_LTP_ protocol (PAS1) induced strong LTP-*like* plasticity lasting for 30-60min. The effect of a second identical PAS_LTP_ protocol (PAS_2_) critically depended on the time between PAS_1_ and PAS_2_. At 10min, PAS_2_ prolonged the PAS_1_-induced LTP-*like* plasticity. At 30min, PAS_2_ augmented the LTP-*like* plasticity induced by PAS_1_, by increasing both magnitude and duration. At 60min and 180min, PAS_2_ had no effect on corticospinal excitability. The cumulative LTP-*like* plasticity after PAS_1_ and PAS_2_ at 30min exceeded significantly the effect of PAS_1_ alone, and the cumulative PAS_1_ and PAS_2_ effects at 60min and 180min. In summary, consecutive PAS_LTP_ protocols interact in human M1 in a time-dependent manner. If spaced by 30min, two consecutive PAS_LTP_ sessions can augment LTP-*like* plasticity in human M1. Findings may inspire further research on optimized therapeutic applications of non-invasive brain stimulation in neurological and psychiatric diseases.

## Introduction

Motor rehabilitation after cerebral injury such as stroke depends on neural plasticity, including synaptic strengthening by long-term potentiation (LTP) [1–4]. Paired associative stimulation (PAS_LTP_) of the human primary motor cortex (M1) can induce an increase in corticospinal excitability as measured by motor evoked potentials (MEPs) beyond the stimulation period [5], which resembles LTP as studied at the cellular level [6–9]. However, LTP-*like* plasticity induced by PAS_LTP_ is regulated by homeostatic metaplasticity [6, 10, 11], i.e., a higher-order form of plasticity, which keeps neuronal and network excitability in a physiological range [12–15]. This homeostatic regulation implies that repeated induction of LTP-*like* plasticity at short delays is suppressed, which may limit the therapeutic potential of PAS_LTP_ to increase corticospinal excitability.

Experiments in animals, however, show that the brain possesses powerful mechanisms, which permit continued synaptic strengthening in the context of prior LTP. One such mechanism is based on an N-methyl-D-aspartate receptor (NMDAR)-dependent form of metaplasticity by which continued synaptic strengthening is possible through activation of metabotropic glutamate receptors (mGluRs) [16]. In addition, inhibition of glycogen synthase kinase-3 beta (GSK3β) results in a ~60 min lasting blockade of subsequent induction of NMDAR-dependent long-term depression (LTD), because expression of LTD requires a high level of GSK3β activity [17]. These mechanisms ensure that information encoded by LTP is not erased during ongoing neural activity, but can be retained. On the system level of the human cortex it has been shown, that motor learning immediately following PAS_LTP_ is not suppressed, as would be expected in the framework of homeostatic metaplasticity, but rather facilitated [18, 19]. In addition, several other studies have occasionally reported non-homeostatic metaplasticity between two consecutive non-invasive brain stimulation protocols, mainly at short intervals of 30min or less [20–24], for review [25]. Even though these studies provide system-level evidence for non-homeostatic interactions between plasticity-inducing non-invasive brain stimulation protocols, and motor learning, respectively, the conditions favoring non-homeostatic vs. homeostatic metaplasticity in the human brain remain poorly understood.

The present study investigated the role of time between two consecutive PAS_LTP_ protocols for repetitive induction of LTP-*like* plasticity in M1 of healthy human subjects. We studied the interactions between two identical sessions of PAS_LTP_ spaced at inter-PAS_LTP_ intervals (IPI) of 10, 30, 60 and 180min. Findings show that metaplasticity in human M1 is expressed in a time-dependent manner with a window of non-homeostatic metaplasticity at an IPI of 30min. If spaced by 30min, two consecutive PAS_LTP_ sessions can augment LTP-*like* plasticity in human M1. These findings may inspire further research on optimized therapeutic applications of non-invasive brain stimulation techniques in a clinical setting.

## Materials and Methods

### Subjects

Written informed consent was obtained from all subjects prior to participation. None of the subjects had a history of neurological or psychiatric disease or was on CNS-active drugs at the time of the experiments and all subjects were checked for contraindications to transcranial magnetic stimulation (TMS) [26]. The study conformed to the Declaration of Helsinki and was approved by the ethics committee of the Medical Faculty of Goethe-University Frankfurt. Twenty-seven subjects were screened for a significant PAS_LTP_-induced increase in motor evoked potential (MEP) amplitude ≥ 1.1 (ratio of mean MEP amplitude post-PAS_LTP_ / pre-PAS_LTP_) [27, 28]. Sixteen subjects fulfilled this criterion (PAS_LTP_ responders) and were included into the study. Of those, four subjects withdrew consent after the first experiment for experiencing some discomfort by TMS. Thus, complete datasets were obtained in 12 subjects (6 females, mean (± SEM) age, 25.6 ± 1.4 years). All subjects were right-handed according to the Edinburgh handedness questionnaire [29].

### Electromyography (EMG) recordings

Surface EMG recordings were obtained from the right abductor pollicis brevis (APB) muscle using Ag-AgCl electrodes in a belly-tendon montage. The raw EMG signal was amplified and band-pass filtered (20Hz–2kHz, Counterpoint Mk2 electromyograph, Dantec, Denmark), digitized at an A/D rate of 5kHz (Micro1401, Cambridge Electronic Design, UK) and stored in a laboratory computer for online display and offline analysis, using customized software (Spike2 for Windows, Version 3.05, Cambridge Electronic Design).

### Transcranial magnetic stimulation (TMS)

Subjects were seated comfortably in a reclining chair. TMS was performed using a Magstim 200 magnetic stimulator (Magstim Company, UK) with a monophasic current waveform. Stimuli were applied to the hand area of left M1 through a figure-of-eight coil (inner diameter of each wing, 70mm) with the handle pointing backwards and 45° away from midline. The optimal coil position for eliciting MEPs in the right APB was marked with a soft-tipped pen on the scalp in order to ensure constant placement of the coil throughout the experiment.

**Fig 1:**
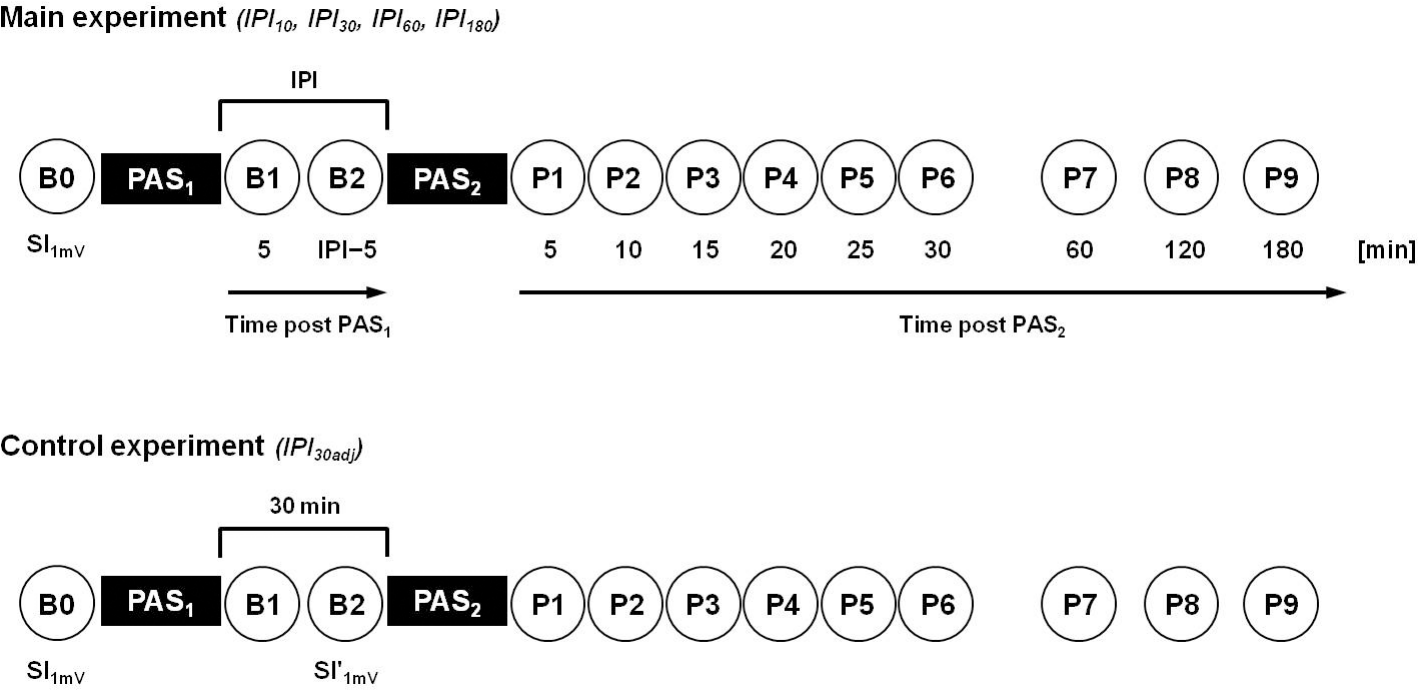
Time line of experiments. In the main experiment, motor-evoked potentials (MEPs) were recorded at all time points (circles; B0, B1-B2, P1-P9) with SI_1mV_, i.e. the stimulation intensity that evoked MEPs of, on average, 1mV peak-to-peak amplitude in the resting *abductor pollicis brevis* muscle at baseline (time point B0). Note that subjects took part in four experimental sessions in a crossover design with different intervals between the consecutive sessions of two identical LTP-*like* plasticity inducing PAS_LTP_ protocols (PAS_1_, PAS_2_): 10min (IPI_10_), 30min (IPI_30_), 60min (IPI_60_), and 180min (IPI_180_). In the control experiment (IPI_30adj_) SI_1mV_ was readjusted at time point B2 to match baseline MEPs of 1mV peak-to-peak amplitude (SI'_1mV_). Otherwise, the control experiment was identical to the IPI_30_ condition of the main experiment.

At the beginning of each experiment the resting motor threshold (RMT) was measured, which was defined as the lowest intensity (indicated in percent of maximum stimulator output, MSO) that elicited small MEPs (>50 μV) in at least five out of ten consecutive trials in the resting APB. RMT was determined to nearest 1% of MSO. Thereafter, the intensity to elicit MEPs of, on average, 1mV peak-to-peak amplitude (SI_1mV_) was determined in the resting APB. This stimulus intensity was then kept constant throughout a given session of the main experiment (**Fig 1**).

### Paired associative stimulation (PAS_LTP_)

PAS_LTP_ was applied according to a protocol originally described by Stefan and colleagues [5] and slightly modified by our group [6, 10]. Briefly, electrical stimulation of the median nerve at the wrist of the right hand was applied through a bipolar electrode (cathode proximal), using constant-current square-wave pulses (duration, 1ms) at an intensity of three times the perceptual sensory threshold. Each stimulus was followed by single-pulse TMS of the left M1 at SI_1mV_. The interstimulus interval equaled the individual N20-latency of the median nerve somatosensory-evoked cortical potential plus 2ms (mean ± SEM, 21.3 ± 0.3ms). At this or similar intervals PAS induces an LTP-*like* increase of MEPs in the majority of subjects [5, 6, 27, 30–32]. PAS_LTP_ consisted of 225 stimulus pairs applied at a frequency of 0.25Hz. The level of attention, a significant modulator of PAS_LTP_ effects [33], was controlled and attention was maximized to the stimulated hand by a light emitting diode (LED) attached to the right wrist which flashed randomly (0.2–1Hz) during PAS_LTP_. Subjects were requested to count and report the total number of flashes as correctly as possible at the end of PAS_LTP_.

### Experimental design

In the main experiment, all subjects took part in four different sessions in a pseudorandomized crossover design (**Fig 1**). Each session consisted of two identical, consecutive PAS_LTP_ protocols (PAS_1_, PAS_2_) with a specific inter-PAS_LTP_ interval (IPI). Intervals were 10min (IPI_10_), 30min (IPI_30_), 60min (IPI_60_) and 180min (IPI_180_). Between PAS_1_ and PAS_2_ subjects were requested to stay awake, keep seated and not to use their stimulated hand. The order of IPI conditions was pseudo-randomized across subjects and experimental sessions were separated by at least three days to avoid carry-over effects (mean individual minimum inter-session interval 7.6 days, range 3–14 days).

**Fig 2:**
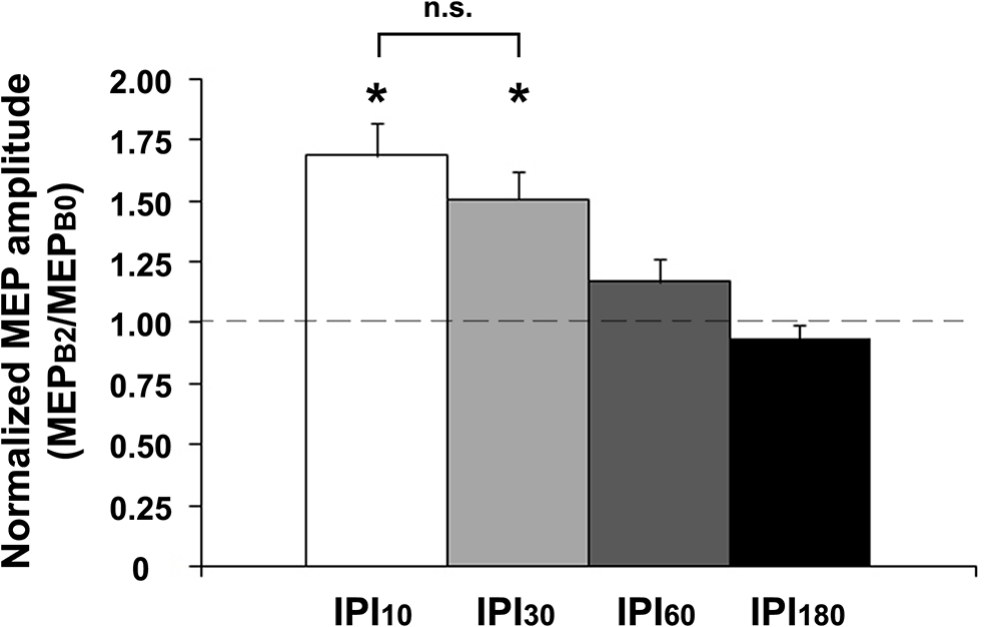
PAS_1_-induced increases in MEP amplitude. PAS_1_ resulted in comparable immediate MEP amplitude increases (MEP_B1_/MEP_B0_) in all IPI conditions (data not shown). At time point B2 (MEP_B2_/MEP_B0_), MEP amplitude increases were present 10min (IPI_10_) and 30min (IPI_30_) after PAS_1_, but no longer at 60min (IPI_60_) and 180min (IPI_180_) after PAS_1_. Asterisks indicate significant differences from 1 (*P* < 0.01; one-sample two-tailed *t* tests). Note that MEP_B2_/MEP_B0_ was not significantly different between conditions IPI_10_ and IPI_30_ (*P* > 0.09; paired two-tailed *t* tests). Data are means ± 1 SEM from twelve subjects.

**Fig 3:**
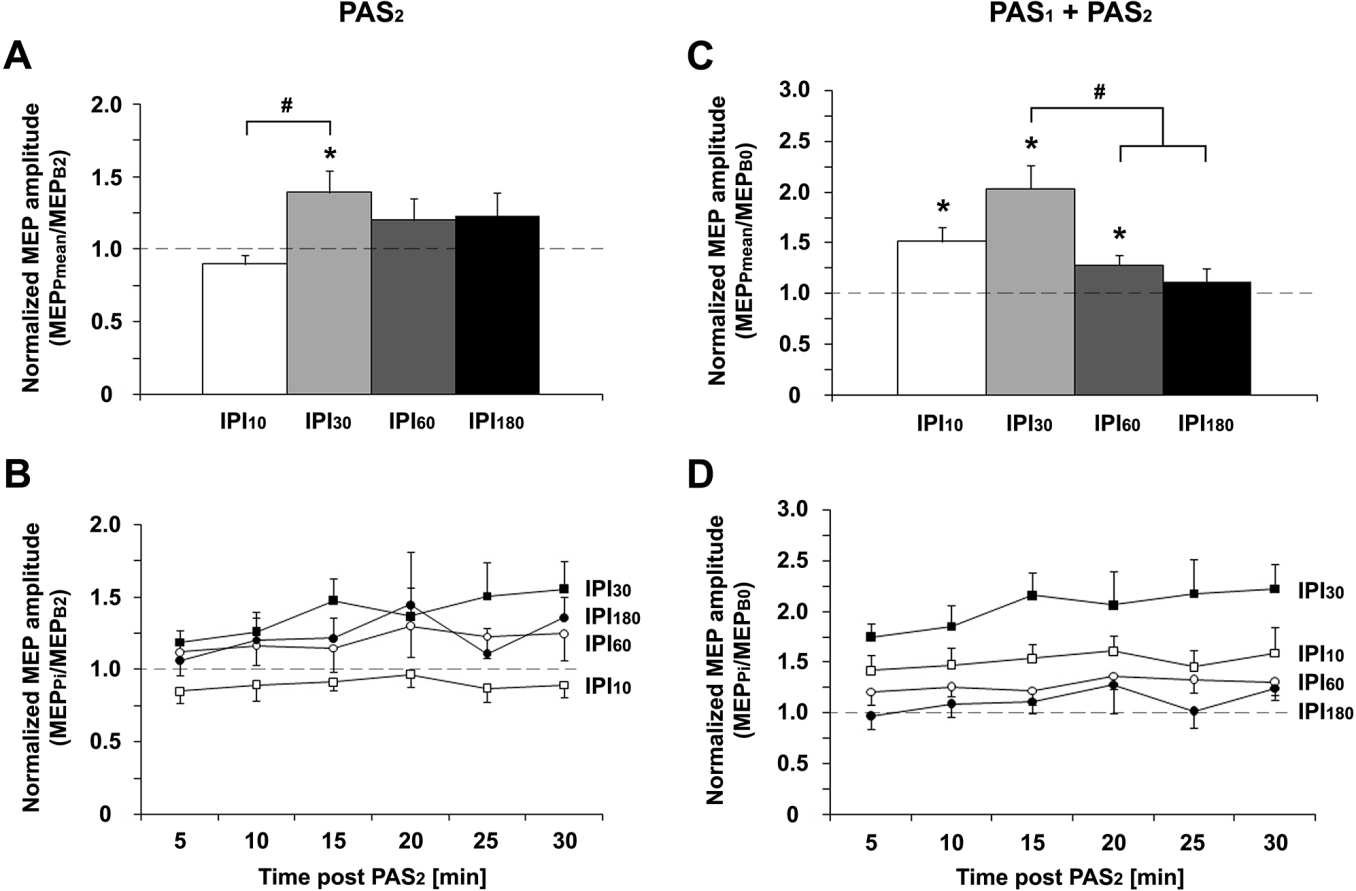
PAS_2_-induced increases in MEP amplitude after PAS_1_-priming. **A**: The PAS_2_-induced increase in MEP amplitude after PAS_1_-priming (MEP_Pmean_/MEP_B2_) was significantly higher at IPI_30_ than IPI_10_ (#, *P* < 0.05; paired two-tailed *t* test). **B**: Time course of MEP_Pi_/MEP_B2_ (i = 1,2,…,6) over 30min after PAS_2_ for IPI_10_ (open squares), IPI_30_ (filled squares), IPI_60_ (open circles), and IPI_180_ (filled circles). **C**: The cumulative effect of PAS_1_ and PAS_2_ on MEP amplitudes (MEP_Pmean_/MEP_B0_) was significantly higher for IPI_30_ than for IPI_60_ and IPI_180_ (#, *P* < 0.05; paired two-tailed *t* tests), and showed a trend to be higher for IPI_30_ vs. IPI_10_ (*P* = 0.095; paired two-tailed *t* test). **D**: Time course of MEP_Pi_/MEP_B2_ (i = 1,2,…,6) over 30min after PAS_2_ for IPI_10_ (open squares), IPI_30_ (filled squares), IPI_60_ (open circles), and IPI_180_ (filled circles). *, *P* < 0.05; one-sample two-tailed *t* tests. Data are means ± 1 SEM from twelve subjects.

To test whether the significant PAS_2_-induced increase of MEP amplitude in the IPI_30_ condition could be attributed to the PAS_1_-induced increase in MEP amplitude at time point B2 immediately before PAS_2_ (see below and cf. **Figs 2** and **3**), we conducted a control experiment (IPI_30adj_), in which we readjusted MEP amplitudes at time point B2 by reducing the stimulation intensity (SI'_1mV_) in order to match baseline MEPs of, on average, 1mV peak-to-peak amplitude. The readjusted stimulation intensity SI'_1mV_ was then used for PAS_2_ and all following MEP measurements. Otherwise the control experiment was identical to the IPI_30_ condition of the main experiment (**Fig 1**). Nine subjects (4 females, mean (± SEM) age, 26.1 ± 1.6 years) took part in the control experiment.

### Quantification of PAS_LTP_ effects

The PAS_1_ effect was quantified by comparing 20 single-trial peak-to-peak MEP amplitudes at SI_1mV_ 5min after PAS_1_ (time point B1) with those at baseline immediately before PAS_1_ (time point B0; **cf. Fig 1**). The inter-trial interval was 10s ± 25% to limit anticipation of the next trial. If the PAS_1_ effect (ratio MEP_B1_/MEP_B0_) was < 1.1, the experiment was stopped, the data were discarded and the experiment was repeated on another day, to minimize intra-individual variability [34] and ensure PAS_1_-induced LTP-*like* plasticity in all experiments (overall, 12 repeated experiments in all subjects; 1.0 ± 0.4 repeated experiments per subject). Five minutes before the second PAS_LTP_ intervention (PAS_2_) another block of 20 MEPs was measured (time point B2; in case of IPI_10_ B1 was taken as B2). After PAS_2_ six blocks of MEP measurements were performed at intervals of 5min (time points P1–P6) to monitor PAS_2_ effects on MEPs for 30min after the end of PAS_2_. In six subjects, additional MEP measurements were conducted 60min (time point P7), 120min (P8), and 180min (P9) after PAS_2_ to determine the time course of the return of PAS_2_-induced MEP amplitude increases (see Results) back to baseline. All measurements were recorded in the resting APB at SI_1mV_, except for the control experiment (IPI_30adj_), in which the TMS intensity was readjusted to SI'_1mV_ at time point B2 and then used for PAS_2_ and all following MEP measurements. Complete voluntary relaxation was monitored by audio-visual feedback of the EMG raw signal at high gain (at 50μV ⁄ division). Trials contaminated with voluntary EMG activity were excluded from analysis (<1% of all trials).

### Statistical analyses

Statistical testing was performed with IBM SPSS Statistics (Version 20.0.0). To test for differences of RMT, SI_1mV_, and MEP amplitude at baseline (time point B0) independent one-way repeated measures analyses of variance (rmANOVAs) with the within-subject factor IPI (IPI_10_, IPI_30_, IPI_60_, IPI_180_) were conducted. To test for differences of the PAS_1_ effect one-way rmANOVAs with the within-subject factor IPI (IPI_10_, IPI_30_, IPI_60_, IPI_180_) were performed separately on mean MEP amplitudes at time points B1 and B2, respectively, normalized to the individual mean MEP amplitude at time point B0 (MEP_B1_/MEP_B0_; MEP_B2_/MEP_B0_). To test for the effects of IPI (IPI_10_, IPI_30_, IPI_60_, IPI_180_) and TIME (P1–P6) on PAS_2_ effects, two-way rmANOVAs were performed separately on mean MEP amplitudes at time points P1–P6 normalized to the individual mean MEP amplitude at time points B0 and B2, respectively (MEP_Pi_/MEP_B0,_ MEP_Pi_/MEP_B2_, i = 1,2,…,6). Normalization to B2 provides information specifically on the effects of PAS_2_ while normalization to B0 provides information on the cumulative effects of PAS_1_ and PAS_2_.

Mauchly’s test was applied to test for non-sphericity and in case of violation of sphericity the Greenhouse-Geisser correction was used. Conditional on significant main effects or their interactions in the rmANOVAs, *post hoc* pairwise comparisons or one-sample two-tailed *t* tests were performed. The Bonferroni correction was applied to adjust for multiple comparisons.

To compare the time course of PAS_1_- and PAS_2_-induced changes in MEP amplitude, the MEP amplitude raw data after PAS_1_ and PAS_2_ were fitted independently with a linear mixed effects model with fractional polynomials at all IPIs. PAS_1_ data comprised mean MEP amplitudes measured at time points B0 (time: 0min) and B2 across IPI conditions (time: 10min at IPI_10_, 30min at IPI_30_, 60min at IPI_60_, and 180min at IPI_180_), whereas PAS_2_ data comprised mean MEP amplitudes measured at time points B2 (time: 0min), P2 (time: 10min), P6 (time: 30min), P7 (time: 60min), and P9 (time: 180min), respectively, for each IPI condition separately. Model functions for PAS_1_- and PAS_2_ effects on MEP amplitudes were compared using *F*- and *t*-statistics, respectively.

In all tests the significance level was set to *P* < 0.05. All data are expressed as means ± SEM.

## Results

All subjects tolerated the experimental procedures well without any adverse effects.

### Baseline excitability data (RMT, SI_1mV_, MEP)

The data are summarized in **Table 1**. There were no significant differences between IPI conditions on RMT (*F*_3,33_ = 0.55, *P* = 0.53), SI_1mV_ (*F*_3,33_ = 0.46, *P* = 0.71), or MEP amplitude (*F*_3,33_ = 1.07, *P* = 0.38) at baseline (time point B0).

**Table 1:** Summary of baseline excitability measures in the different IPI conditions.

Condition	RMT	SI_1mV_	MEP
IPI_10_	40.1 ± 2.1	50.8 ± 2.2	0.97 ± 0.04
IPI_30_	39.3 ± 1.6	51.4 ± 2.0	0.91 ± 0.05
IPI_60_	38.9 ± 1.8	50.5 ± 2.4	0.93 ± 0.04
IPI_180_	38.8 ± 1.4	49.6 ± 1.7	1.02 ± 0.05

Abbreviations: IPI, inter-PAS_LTP_ interval [in min, index]; MEP, motor-evoked potential [in mV]; RMT, resting motor threshold [in %maximum stimulator output, MSO]; SI_1mV_, stimulation intensity that induces MEPs of 1mV peak-to-peak amplitude on average [in %MSO].

### PAS_1_ effects on MEP amplitudes (comparison of time points B1 and B2 vs. B0)

The one-way rmANOVA for the PAS_1_ effect at time point B1 (MEP_B1_/MEP_B0_) showed no significant difference between IPI conditions (*F*_3,33_ = 0.71, *P* = 0.55), indicating similar PAS_1_-induced LTP-*like* plasticity across all IPI conditions. MEP_B1_/MEP_B0_ was > 1.0 for IPI_10_ (1.69 ± 0.13; *t* = 5.25, *P* = 0.0003), IPI_30_ (1.49 ± 0.10; *t* = 5.01, *P* = 0.0004), IPI_60_ (1.50 ± 0.07; *t* = 7.57, *P* < 0.0001), and IPI_180_ (1.53 ± 0.13; *t* = 4.12, *P* = 0.0017). In contrast, the one-way rmANOVA for the PAS_1_ effect at time point B2 (MEP_B2_/MEP_B0_) immediately before PAS_2_ showed a significant effect of IPI (*F*_3,33_ = 11.74, *P* < 0.001). MEP_B2_/MEP_B0_ was > 1.0 for IPI_10_ (1.69 ± 0.13; *t* = 5.25, *P* = 0.0003) and IPI_30_ (1.51 ± 0.12; *t* = 4.32, *P* = 0.0012), but no longer for IPI_60_ (1.17 ± 0.09; *t* = 1.90, *P* = 0.084) or IPI_180_ (0.94 ± 0.05; *t* = -1.20, *P* = 0.26) (**Fig 2**). Of note, there was no significant difference of MEP_B2_/MEP_B0_ between conditions IPI_10_ and IPI_30_ (*P* > 0.9), indicating similar persistence of PAS_1_-induced LTP-*like* plasticity in these conditions at time point B2 immediately before PAS_2_.

### PAS_2_ effects on MEP amplitudes (comparison of time points P1-P6 vs. B2)

The two-way rmANOVA for the PAS_2_ effect on MEP amplitudes at time points P1–P6 (MEP_Pi_/MEP_B2_, i = 1,2,…,6) showed a significant effect of IPI (*F*_3,33_ = 2.94, *P* = 0.048) and TIME (*F*_5,55_ = 2.50, *P* = 0.041), whereas the interaction IPI with TIME was not significant (*F*_15,165_ = 0.73, *P* = 0.75) (**Fig 3A and 3B**). MEP_Pmean_/MEP_B2_ was > 1.0 for IPI_30_ (1.39 ± 0.15; *t* = 2.59, *P* = 0.025), but not for IPI_10_ (0.90 ± 0.06; *t* = -1.72, *P* = 0.11), IPI_60_ (1.20 ± 0.15; *t* = 1.34, *P* = 0.21) or IPI_180_ (1.23 ± 0.16; *t* = 1.48, *P* = 0.17). *Post hoc* testing showed that MEP amplitudes after PAS_2_ (MEP_Pmean_/MEP_B2_) were significantly different between conditions IPI_10_ and IPI_30_ (*P* = 0.047), while all other pairwise comparisons were not significant (all *P* > 0.2).

**Fig 4:**
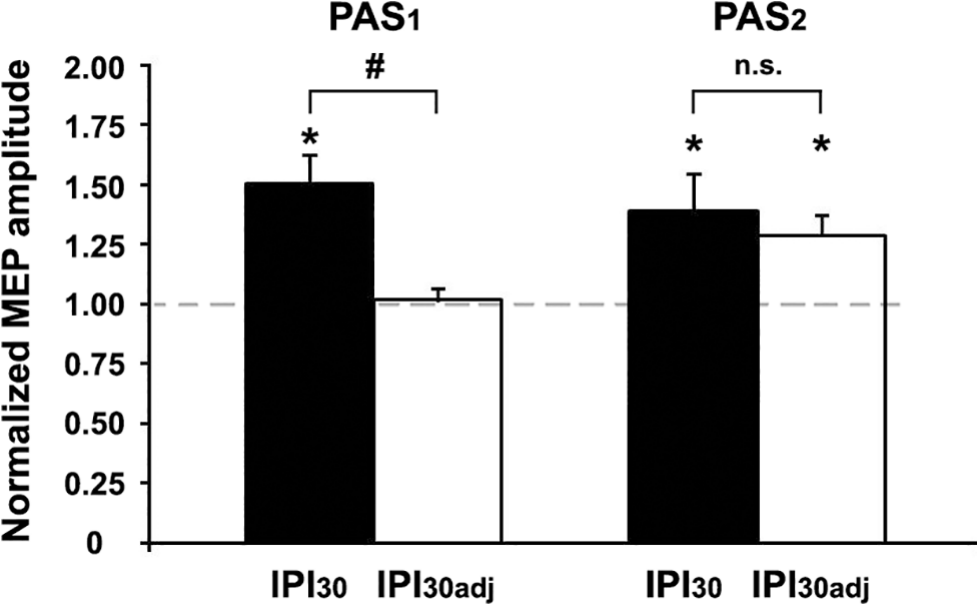
PAS_2_-induced increase in MEP amplitude after PAS_1_-priming in the control experiment (IPI_30adj_). MEP amplitudes at time point B2 immediately before PAS_2_ were successfully readjusted by reducing the stimulation intensity (SI'_1mV_) to match baseline MEPs at time point B0 (PAS_1_, MEP_B2/B0_). Despite this readjustment, PAS_2_ induced a similar increase in MEP amplitudes in the control (IPI_30adj_) compared to the main experiment (IPI_30_) (PAS_2_, MEP_Pmean_/MEP_B2_). *, *P* < 0.05, one-sample two-tailed *t* tests; #, *P* < 0.01, unpaired two-tailed *t* test. Data from the control experiment are from nine subjects, means ± 1 SEM.

It could be argued that the significant PAS_2_ effect on MEP amplitudes at IPI_30_ was due to the increased MEP amplitude at time point B2 immediately before PAS_2_ (cf. **Fig 2**). To address this issue, we conducted a control experiment (IPI_30adj_), in which we readjusted MEP amplitudes at time point B2 immediately before PAS_2_ by reducing the stimulation intensity (SI'_1mV_) in order to match baseline MEPs (0.99 ± 0.05mV at time point B0 with SI_1mV_ = 45.8 ± 1.6%MSO; 1.01 ± 0.06mV at time point B2 with SI'_1mV_ = 44.2 ± 1.6%MSO, *t* = -0.29, *P* > 0.7; paired two-tailed *t* test). Thus, whilst PAS_LTP_ induced similar MEP increases at time point B1 (MEP_B1_/MEP_B0_) in the control (IPI_30adj_, 1.31 ± 0.05; *t* = 6.62, *P* = 0.0002, one-sample two-tailed *t* test) and the main experiment (IPI_30_, 1.49 ± 0.10; *t* = 5.01, *P* = 0.0004, one-sample two-tailed *t* test; IPI_30adj_ vs. IPI_30:_ *P* > 0.15, unpaired two-tailed *t* test), MEP_B2_/MEP_B0_ was significantly different between the two experiments (IPI_30adj_: 1.02 ± 0.04, *t* = 0.38, *P* > 0.7; IPI_30_: 1.51 ± 0.12; *t* = 4.32, *P* = 0.0012, one-sample two-tailed *t* tests; IPI_30adj_ vs. IPI_30:_ *P* = 0.0025, unpaired two-tailed *t* test; **Fig 4**). However, PAS_2_ induced a similar increase in MEP amplitudes (MEP_Pmean_/MEP_B2_) in the control (IPI_30adj_: 1.29 ± 0.09, *t* = 3.09, *P* = 0.015) compared to the main experiment (IPI_30_: 1.39 ± 0.15; *t* = 2.59, *P* = 0.025; one-sample two-tailed *t* tests; IPI_30adj_ vs. IPI_30:_ *P* > 0.5, unpaired two-tailed *t* test; **Fig 4**). This finding strongly suggested that the increased MEP amplitude at time point B2 at IPI_30_ was not relevant for the significant PAS_2_-induced increase of MEP amplitudes in this condition_._ This notion was further supported by the observation, that PAS_2_ had significantly different effects on MEP amplitudes at IPI_30_ vs. IPI_10_ in the main experiment (cf. **Fig 3A and 3B**), although MEP amplitudes were increased to a similar level immediately before PAS_2_ (MEP_B2_/MEP_B0_) in these two conditions (cf. **Fig 2**).

### Cumulative PAS_1_ and PAS_2_ effects on MEP amplitudes (comparison of time points P1-P6 vs. B0)

The two-way rmANOVA for the cumulative PAS_1_ and PAS_2_ effect on MEP amplitudes at time points P1–P6 (MEP_Pi_/MEP_B0_, i = 1,2,…,6) showed a significant effect of IPI (*F*_3,33_ = 11.36, *P* < 0.0001) and TIME (*F*_5,55_ = 2.49, *P* = 0.042), whereas the interaction IPI with TIME was not significant (*F*_15,165_ = 0.66, *P* = 0.82) (**Fig 3C and 3D**). MEP_Pmean_/MEP_B0_ was > 1.0 for IPI_10_ (1.51 ± 0.14; *t* = 3.61, *P* = 0.0041), IPI_30_ (2.04 ± 0.23; *t* = 4.54, *P* = 0.0009), and IPI_60_ (1.28 ± 0.09; *t* = 2.98, *P* = 0.013), but not for IPI_180_ (1.12 ± 0.12; *t* = 0.97, *P* = 0.35). *Post hoc* testing showed significantly higher MEP amplitudes after PAS_2_ (MEP_Pmean_/MEP_B0_) for IPI_30_ than for IPI_60_ (*P* = 0.009) and IPI_180_ (*P* = 0.01), and a trend for higher MEP amplitudes for IPI_30_ vs. IPI_10_ (*P* = 0.095). Notably, at IPI_30_ the MEP amplitude increase 30 minutes after PAS_2_ normalized to baseline (MEP_P6_/MEP_B0_ = 2.22 ± 0.25; **Fig 3D**) was significantly higher than the MEP amplitude increase 30 minutes after PAS_1_ in this condition (MEP_B2_/MEP_B0_ = 1.51 ± 0.12; *P* = 0.018, paired two-tailed *t* test), indicating that consecutive application of PAS_1_ and PAS_2_ at IPI_30_ induced significantly higher MEP increases than application of PAS_1_ alone.

### Modelling of PAS_1_ and PAS_2_ effects on MEP amplitudes

**Fig 5:**
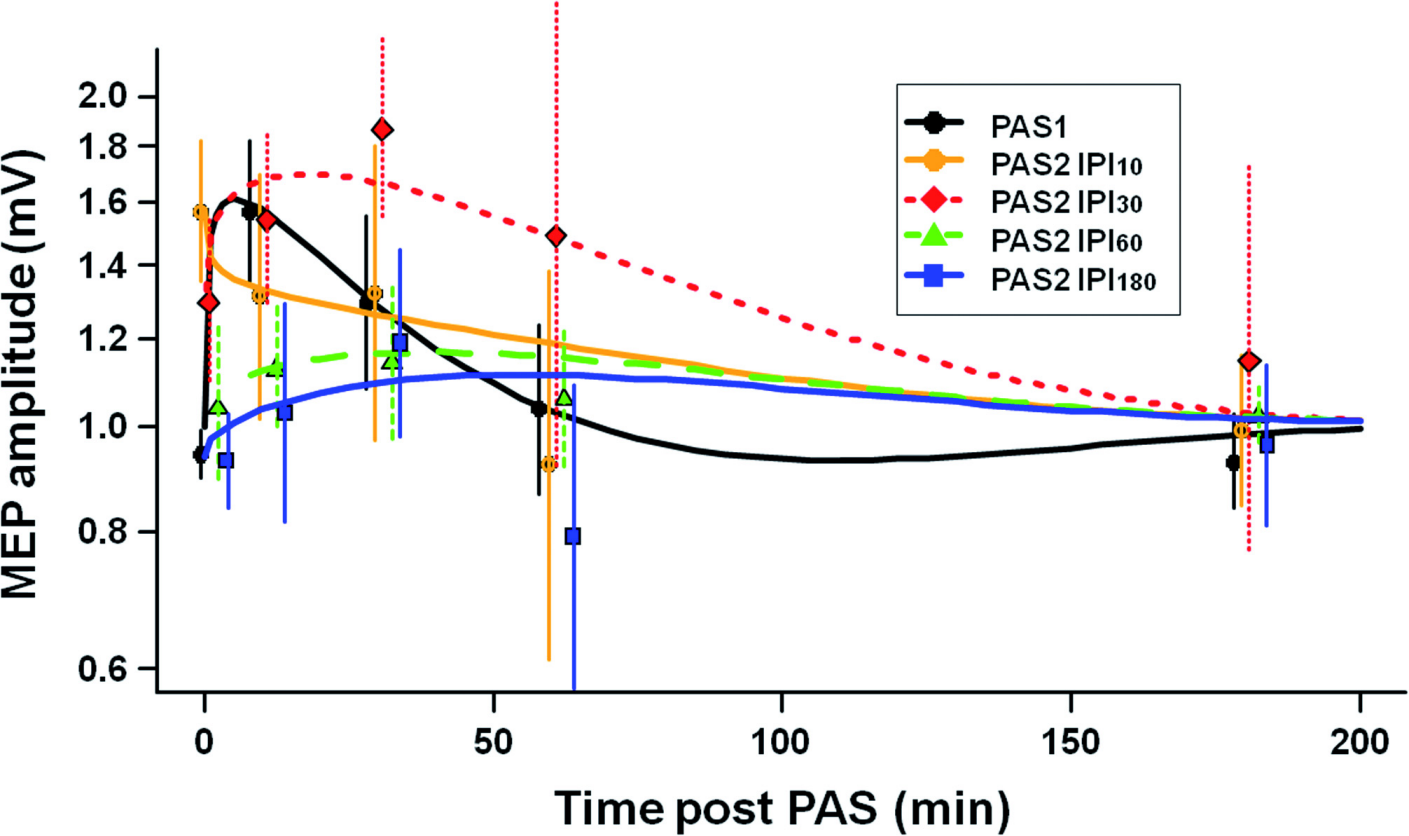
Computational modeling of the time course of PAS_1_ and PAS_2_ effects. MEP amplitudes as a function of time modeled from the experimental MEP raw data independently for PAS_1_ and PAS_2_ at all IPIs. PAS_1_ and PAS_2_ model functions, i.e. the time course of MEP amplitude changes after PAS_1_ vs. those after PAS_2_, were significantly different at all IPIs (*P* < 0.0001 each). In addition, PAS_2_ effects at IPI_10_ and IPI_30_ were significantly different from PAS_2_ effects at IPI_60_ and IPI_180_ (*P* < 0.0001), and PAS_2_ effects at IPI_10_ from PAS_2_ effects at IPI_30_ (*P* = 0.033), but not PAS_2_ effects at IPI_60_ from those at IPI_180_ (*P* > 0.5). Experimental data are shown as mean ± SEM. Note that data for PAS_2_ at time points 60min and 180min post PAS_2_ are from six subjects only, whereas all other data are from twelve subjects (see Material and Methods). Y-axis, logarithmic scaling.

Computational modelling of the PAS_1_ and PAS_2_ effects on the absolute MEP amplitude raw data as a function of time revealed highly significant differences between PAS_1_ and PAS_2_ MEP functions (*P* < 0.0001) (**Fig 5**). *Post hoc* testing showed significant differences between the PAS_1_ and PAS_2_ model functions for all IPI conditions (*P* < 0.0001 each), suggesting that PAS_2_ effects on MEP amplitudes were modulated by prior application of PAS_1_ in all IPI conditions. Specifically, at IPI_10_ PAS_2_ prolonged the MEP increase induced by PAS_1_. At IPI_30_, PAS_2_ induced an extra MEP increase, notably with a longer time course as compared to the PAS_1_-induced MEP increase. In contrast, at IPI_60_ and IPI_180_ there was no significant change in MEP amplitudes after PAS_2_.

In addition, the time courses of PAS_2_-induced MEP amplitude changes were significantly different between IPI conditions (*P* < 0.0001). *Post hoc* analysis showed that PAS_2_ effects at IPI_10_ and IPI_30_ were significantly different from PAS_2_ effects at IPI_60_ and IPI_180_ (all *P* < 0.0001), and PAS_2_ effects at IPI_10_ were significantly different from PAS_2_ effects at IPI_30_ (*P* = 0.033), whereas the comparison of PAS_2_ effects at IPI_60_ and IPI_180_ showed no significant differences (*P* > 0.5). These differences are explained by a prolonged MEP increase at IPI_30_ compared to all other conditions (**Fig 5**).

## Discussion

We showed here that LTP-*like* plasticity can be augmented in human M1 when two consecutive PAS_LTP_ protocols are spaced by 30min. In contrast, at longer intervals (60-180min) we found a suppressive interaction between two consecutive PAS_LTP_ protocols. These findings support the notion of non-homeostatic and homeostatic metaplasticity, respectively, and will be discussed in detail below.

The PAS_1_-induced MEP increase lasted for 30-60min, in accord with the literature, and represents a form of plasticity resembling LTP as studied at the cellular level [5, 7, 9, 35]. In contrast, the effects of PAS_2_ on MEP amplitude depended critically on the interval to PAS_1_. Computational modelling of the time courses of MEP amplitude (**Fig 5**) revealed significant differences between PAS_1_ and PAS_2_ effects on MEP amplitude for all IPI conditions, indicating that priming M1 by PAS_1_ modulated the effects of a subsequent identical PAS_2_ protocol for at least three hours.

Metaplasticity constitutes a higher-order form of plasticity, which regulates the magnitude and duration of synaptic plasticity in an activity-dependent manner [12]. Importantly, it modulates the plasticity state of neurons and networks, i.e. the induction of LTP subsequent to the priming stimulation, in the absence of synaptic plasticity induced by the priming stimulation itself. Here, we demonstrated a homeostatic interaction between PAS_1_ and PAS_2_ at IPI_60_ and IPI_180_ (**Fig 3**) in the absence of any persistent PAS_1_-induced MEP increase at the time of PAS_2_ (**Fig 2**). Further, both magnitude and/or duration of PAS_2_-induced LTP-*like* plasticity were modulated by PAS_1_ (**Fig 5**). Thus, these findings support the notion that the interactions between PAS_1_ and PAS_2_ effects described in the present study represent a form of metaplasticity similar to the ones reported at the cellular level [12, 36].

Previous studies have reported on metaplasticity between two consecutive identical non-invasive brain stimulation protocols in the human M1 [10, 20–24, 37–40]. The predominant interaction was homeostatic metaplasticity, but several occasions of non-homeostatic metaplasticity have also been reported, predominantly at short IPIs of 3-20min (for review, [25]). This non-homeostatic metaplasticity resulted in *late* LTD- and LTP-*like* changes in corticospinal excitability that were prevented by pharmacological blockade of NMDA receptors by dextromethorphan [24] and that were resistant to de-depression interventions such as voluntary contraction of the target muscle [22]. These data are compatible with the finding in the present study that two consecutive PAS_LTP_ protocols, if spaced by 30min, resulted in non-homeostatic augmentation of LTP-*like* plasticity with a prolonged duration.

These time-dependent interactions between PAS_1_ and PAS_2_ may be explained in the framework of a cascade model of synaptic plasticity [41]. As synapses are modified during the course of LTP, they change between *discrete* mechanistic states [42–44]. For example, silent synapses, i.e. synapses containing NMDARs, but being devoid of α-amino-3-hydroxy-5-methyl-4-isoxazolepropionic acid receptors (AMPARs) move to a “recently silent state” (i.e. with inserted AMPARs), in which they initially cannot be further potentiated. Additional potentiation is possible only if they move to the “active state”, which occurs at around 30min after LTP induction [42, 45]. Recent results from organotypic entorhino-hippocampal slice cultures suggest that repetitive magnetic stimulation indeed may lead to an activation of silent synapses [46]. As PAS_1_ and PAS_2_ were identical in our study and thus presumably modified the same set of synapses, such a cascade model of synaptic plasticity states may explain why PAS_2_ induced significant LTP-*like* plasticity at IPI_30_, but not at IPI_10_. In addition, the MEP increase after PAS_2_ at IPI_30_ may have been enabled by LTP-induced suppression of subsequent LTD. In rat hippocampus NMDAR-dependent LTP inhibits GSK3β, resulting in a ~60 minutes lasting blockade of subsequent NMDAR-dependent LTD induction [17]. In contrast, the suppressive interactions between PAS_1_ and PAS_2_ at IPI_60_ and IPI_180_ are in line with homeostatic metaplasticity [12]. Evidence in support of the notion of a delayed onset of homeostatic metaplasticity comes from animal experiments that showed that an experience-dependent increase in the NR2A/NR2B NMDAR subunit ratio, which is associated with a rightward shift of the synaptic modification threshold that favors induction of LTD over LTP [47, 48], did not occur within the first ~60min after a priming protocol [49].

An alternative explanation for the augmenting PAS_2_ effect on MEPs at IPI_30_ may have been gating [50] by the increased level of cortical excitability immediately prior to PAS_2_ in this condition (time point B2, **Fig 2**). However, this explanation is rather unlikely, as (i) computational modelling of MEP time functions after PAS_2_ showed significant differences between conditions IPI_10_ and IPI_30_ (cf. **Fig 5**), although the PAS_1_-induced MEP increases immediately before PAS_2_ were comparable in these two conditions (cf. **Fig 2**), and (ii) a control experiment (IPI_30adj_), in which we readjusted MEP amplitudes after PAS_1_ immediately before PAS_2_ to match baseline values, found similar PAS_2_ effects as in the main experiment (IPI_30_), in which MEP amplitudes were not readjusted (**Fig 4**).

At IPI_30_, PAS_2_ induced significant MEP increases over and above those induced by PAS_1_. The cumulative PAS_1_ and PAS_2_ increase of MEP amplitude at IPI_30_ significantly exceeded the PAS_1_ effect alone, and the cumulative effects of PAS_1_ and PAS_2_ at IPI_60_ and IPI_180_. These results show that LTP-*like* plasticity can be augmented in human M1, if consecutive PAS_LTP_ protocols are properly spaced within a window of non-homeostatic metaplasticity.

In contrast, homeostatic metaplasticity, i.e. the suppressive interaction between consecutive PAS_LTP_ protocols at longer intervals (IPI_60_ and IPI_180_), may prevent runaway LTP, enabling the human motor network to maintain its modifiability within a useful dynamic range. In line with this notion, dysfunctional homeostatic metaplasticity can result in a deficient control of activity-dependent plasticity, as seems to be the case in task-dependent dystonia [51, 52]. The mechanisms of homeostatic metaplasticity at IPI_60_ and IPI_180_ are as of yet unclear, but may involve Ca^2+^ influx through voltage-gated Ca^2+^ channels [53], or intracellular Ca^2+^ stores [54].

Studies in experimental animals have provided evidence, that an mGluR-dependent form of LTP is retained in the context of NMDAR-dependent increased synaptic strength [16, 55]. Thus, continued experience leads to increased synaptic strength over time, although NMDAR-dependent LTP is occluded during continued neural activity. It could be speculated that LTP-*like* plasticity induced by PAS_1_ vs. PAS_2_ at IPI_30_ in our experiments was due to different underlying physiological mechanisms, i.e. an NMDAR- and an mGluR-dependent form of LTP-*like* plasticity, respectively. This idea is indirectly supported by the following observations: (i) LTP-*like* plasticity induced by a single PAS_LTP_ protocol can be blocked by an NMDAR antagonist [7]; (ii) the PAS_1_-induced LTP-*like* plasticity in the present study was likely saturated as the MEP amplitude increase of ≥1.5 is among the highest reported in the literature [30, 33, 56]; (iii) the time-course of PAS_1_- vs. PAS_2_-induced MEP changes at IPI_30_ in the present study was significantly different (cf. **Fig 5**). Thus, properly timed consecutive application of non-invasive brain stimulation protocols such as PAS_LTP_ may lead to augmentation of LTP-*like* plasticity through recruitment of different physiological mechanisms. Regardless of the underlying mechanisms, our results provide experimental evidence that consecutive applications of PAS_LTP_ can lead to significantly increased LTP-*like* plasticity as compared to a single PAS_LTP_ session.

At first sight, the present data are at variance with those from one previous study of our group where we found homeostatic metaplasticity between two consecutive PAS_LTP_ protocols if spaced by 30min [10]. However, the two studies differ in one important aspect: in this but not the previous study, we deliberately maximized LTP-*like* plasticity induced by PAS_LTP_ by including only those data with a PAS_1_-induced MEP increase ≥1.1, and by ensuring high attention towards the stimulated hand, which is known to facilitate PAS_LTP_-induced LTP-*like* plasticity [33]. As a result, the PAS_1_–induced MEP increase in this study (time point B1, 1.49 ± 0.10, condition IPI_30_) was significantly higher than in the previous study (1.14 ± 0.12; *t* = 2.24, *P* = 0.036; unpaired two-tailed *t* test). Previous work in mice showed that the expression of non-homeostatic metaplasticity critically depended on the level of the priming activity: only during strong but not weak stimuli, potentiated synapses could be further potentiated, specifically by induction of a switch in NMDAR and mGluR properties [16, 55]. Thus, the significantly stronger LTP-*like* plasticity induced by PAS_1_ in this compared to our previous study [10] may explain why we observed augmentation of LTP-*like* plasticity in the present study only.

In summary, the present study demonstrated that LTP-*like* plasticity in the human M1 can be augmented by application of two consecutive identical PAS_LTP_ protocols if spaced by 30min, while homeostatic interaction occurred at intervals of 60-180min. These findings may inspire further research to optimize therapeutic applications of non-invasive brain stimulation in patients with neurological or psychiatric diseases to modify synaptic transmission in their disordered brain networks more effectively than hitherto possible.

## References

[1] Cooke SF, Bliss TV. Plasticity in the human central nervous system. Brain. 2006;129(Pt 7):1659–73. . 16672292 10.1093/brain/awl082

[2] Murphy TH, Corbett D. Plasticity during stroke recovery: from synapse to behaviour. Nature reviews Neuroscience. 2009;10(12):861–72. 10.1038/nrn2735 . 19888284

[3] Rioult-Pedotti MS, Friedman D, Donoghue JP. Learning-induced LTP in neocortex. Science. 2000;290(5491):533–6. 11039938 10.1126/science.290.5491.533

[4] Rosenkranz K, Kacar A, Rothwell JC. Differential modulation of motor cortical plasticity and excitability in early and late phases of human motor learning. J Neurosci. 2007;27(44):12058–66. . 17978047 10.1523/JNEUROSCI.2663-07.2007 PMC6673358

[5] Stefan K, Kunesch E, Cohen LG, Benecke R, Classen J. Induction of plasticity in the human motor cortex by paired associative stimulation. Brain. 2000;123(Pt 3):572–84. 10686179 10.1093/brain/123.3.572

[6] Ziemann U, Ilic TV, Pauli C, Meintzschel F, Ruge D. Learning modifies subsequent induction of LTP-like and LTD-like plasticity in human motor cortex. J Neurosci. 2004;24(7):1666–72. 14973238 10.1523/JNEUROSCI.5016-03.2004 PMC6730462

[7] Stefan K, Kunesch E, Benecke R, Cohen LG, Classen J. Mechanisms of enhancement of human motor cortex excitability induced by interventional paired associative stimulation. The Journal of physiology. 2002;543(Pt 2):699–708. 12205201 10.1113/jphysiol.2002.023317 PMC2290505

[8] Stefan K, Wycislo M, Gentner R, Schramm A, Naumann M, Reiners K, et al Temporary Occlusion of Associative Motor Cortical Plasticity by Prior Dynamic Motor Training. Cereb Cortex. 2006;16(3):376–85. . 15930370 10.1093/cercor/bhi116

[9] Müller-Dahlhaus F, Ziemann U, Classen J. Plasticity resembling spike-timing dependent synaptic plasticity: the evidence in human cortex. Front Syn Neurosci. 2010;2(34):1–11. 10.3389/fnsyn.2010.00034 PMC3059695 21423520

[10] Müller JF, Orekhov Y, Liu Y, Ziemann U. Homeostatic plasticity in human motor cortex demonstrated by two consecutive sessions of paired associative stimulation. The European journal of neuroscience. 2007;25:3461–8. 17553015 10.1111/j.1460-9568.2007.05603.x

[11] Pötter-Nerger M, Fischer S, Mastroeni C, Groppa S, Deuschl G, Volkmann J, et al Inducing homeostatic-like plasticity in human motor cortex through converging cortico-cortical inputs. Journal of neurophysiology. 2009;102(6):3180–90. 10.1152/jn.91046.2008 19726723

[12] Abraham WC. Metaplasticity: tuning synapses and networks for plasticity. Nature reviews Neuroscience. 2008;9(5):387–99. 10.1038/nrn2356 18401345

[13] Abraham WC, Bear MF. Metaplasticity: the plasticity of synaptic plasticity. Trends in neurosciences. 1996;19(4):126–30. . 8658594 10.1016/s0166-2236(96)80018-x

[14] Bienenstock EL, Cooper LN, Munro PW. Theory for the development of neuron selectivity: orientation specificity and binocular interaction in visual cortex. J Neurosci. 1982;2(1):32–48. . 7054394 10.1523/JNEUROSCI.02-01-00032.1982 PMC6564292

[15] Hulme SR, Jones OD, Abraham WC. Emerging roles of metaplasticity in behaviour and disease. Trends in neurosciences. 2013;36(6):353–62. Epub 2013/04/23. 10.1016/j.tins.2013.03.007 . 23602195

[16] Clem RL, Celikel T, Barth AL. Ongoing in Vivo Experience Triggers Synaptic Metaplasticity in the Neocortex. Science. 2008;319:101–4. 10.1126/science.1143808 18174444

[17] Peineau S, Taghibiglou C, Bradley C, Wong TP, Liu L, Lu J, et al LTP inhibits LTD in the hippocampus via regulation of GSK3beta. Neuron. 2007;53(5):703–17. . 17329210 10.1016/j.neuron.2007.01.029

[18] Teo JT, Swayne OB, Cheeran B, Greenwood RJ, Rothwell JC. Human Theta Burst Stimulation Enhances Subsequent Motor Learning and Increases Performance Variability. Cereb Cortex. 2011;21(7):1627–38. 10.1093/cercor/bhq231 21127013

[19] Jung P, Ziemann U. Homeostatic and non-homeostatic modulation of learning in human motor cortex. J Neurosci. 2009;29:5597–604. 10.1523/JNEUROSCI.0222-09.2009 19403826 PMC6665848

[20] Gamboa OL, Antal A, Laczo B, Moliadze V, Nitsche MA, Paulus W. Impact of repetitive theta burst stimulation on motor cortex excitability. Brain stimulation. 2011:in press. 10.1016/j.brs.2010.09.008 21777874

[21] Goldsworthy MR, Pitcher JB, Ridding MC. The application of spaced theta burst protocols induces long-lasting neuroplastic changes in the human motor cortex. The European journal of neuroscience. 2012;35:125–34. 10.1111/j.1460-9568.2011.07924.x 22118241

[22] Goldsworthy MR, Müller-Dahlhaus F, Ridding MC, Ziemann U. Resistant Against De-depression: LTD-Like Plasticity in the Human Motor Cortex Induced by Spaced cTBS. Cereb Cortex. 2014. Epub 2014/02/04. 10.1093/cercor/bht353 . 24488942

[23] Monte-Silva K, Kuo MF, Liebetanz D, Paulus W, Nitsche MA. Shaping the optimal repetition interval for cathodal transcranial direct current stimulation (tDCS). Journal of neurophysiology. 2010;103(4):1735–40. 10.1152/jn.00924.2009 20107115

[24] Monte-Silva K, Kuo MF, Hessenthaler S, Fresnoza S, Liebetanz D, Paulus W, et al Induction of late LTP-like plasticity in the human motor cortex by repeated non-invasive brain stimulation. Brain stimulation. 2013;6(3):424–32. Epub 2012/06/15. 10.1016/j.brs.2012.04.011 . 22695026

[25] Müller-Dahlhaus F, Ziemann U. Metaplasticity in Human Cortex. The Neuroscientist: a review journal bringing neurobiology, neurology and psychiatry. 2015;21(2):185–202. 10.1177/1073858414526645 24620008

[26] Keel JC, Smith MJ, Wassermann EM. A safety screening questionnaire for transcranial magnetic stimulation [letter]. Clin Neurophysiol. 2001;112:720 11332408 10.1016/s1388-2457(00)00518-6

[27] Müller-Dahlhaus JF, Orekhov Y, Liu Y, Ziemann U. Interindividual variability and age-dependency of motor cortical plasticity induced by paired associative stimulation. Experimental brain research Experimentelle Hirnforschung. 2008;187(3):467–75. 10.1007/s00221-008-1319-7 18320180

[28] Heidegger T, Krakow K, Ziemann U. Effects of antiepileptic drugs on associative LTP-like plasticity in human motor cortex. The European journal of neuroscience. 2010;32:1215–22. 10.1111/j.1460-9568.2010.07375.x 20726885

[29] Oldfield RC. The assessment and analysis of handedness: the Edinburgh inventory. Neuropsychologia. 1971;9(1):97–113. 5146491 10.1016/0028-3932(71)90067-4

[30] Wolters A, Sandbrink F, Schlottmann A, Kunesch E, Stefan K, Cohen LG, et al A temporally asymmetric Hebbian rule governing plasticity in the human motor cortex. Journal of neurophysiology. 2003;89(5):2339–45. 12612033 10.1152/jn.00900.2002

[31] Hamada M, Galea JM, Di Lazzaro V, Mazzone P, Ziemann U, Rothwell JC. Two distinct interneuron circuits in human motor cortex are linked to different subsets of physiological and behavioral plasticity. J Neurosci. 2014;34(38):12837–49. 10.1523/JNEUROSCI.1960-14.2014 . 25232119 PMC6705319

[32] Hamada M, Strigaro G, Murase N, Sadnicka A, Galea JM, Edwards MJ, et al Cerebellar modulation of human associative plasticity. The Journal of physiology. 2012;590(10):2365–74. 10.1113/jphysiol.2012.230540 22473780 PMC3424758

[33] Stefan K, Wycislo M, Classen J. Modulation of associative human motor cortical plasticity by attention. Journal of neurophysiology. 2004;92:66–72. . 14724259 10.1152/jn.00383.2003

[34] Fratello F, Veniero D, Curcio G, Ferrara M, Marzano C, Moroni F, et al Modulation of corticospinal excitability by paired associative stimulation: reproducibility of effects and intraindividual reliability. Clin Neurophysiol. 2006;117(12):2667–74. . 17011821 10.1016/j.clinph.2006.07.315

[35] Ziemann U, Paulus W, Nitsche MA, Pascual-Leone A, Byblow WD, Berardelli A, et al Consensus: Motor cortex plasticity protocols. Brain stimulation. 2008;1(3):164–82. 10.1016/j.brs.2008.06.006 20633383

[36] Mockett BG, Hulme SR. Metaplasticity: new insights through electrophysiological investigations. Journal of integrative neuroscience. 2008;7(2):315–36. . 18763726 10.1142/s0219635208001782

[37] Murakami T, Müller-Dahlhaus F, Lu MK, Ziemann U. Homeostatic Metaplasticity of Corticospinal Excitatory and intracortical Inhibitory Neural Circuits in Human Motor Cortex. The Journal of physiology. 2012;590(22):5765–81. Epub 2012/08/30. 10.1113/jphysiol.2012.238519 . 22930265 PMC3528990

[38] Mastroeni C, Bergmann TO, Rizzo V, Ritter C, Klein C, Pohlmann I, et al Brain-derived neurotrophic factor—a major player in stimulation-induced homeostatic metaplasticity of human motor cortex? PloS one. 2013;8(2):e57957 Epub 2013/03/08. 10.1371/journal.pone.0057957 23469118 PMC3585283

[39] Fricke K, Seeber AA, Thirugnanasambandam N, Paulus W, Nitsche MA, Rothwell JC. Time course of the induction of homeostatic plasticity generated by repeated transcranial direct current stimulation (tDCS) of the human motor cortex. Journal of neurophysiology. 2011;105(3):1141–9. 10.1152/jn.00608.2009 21177994

[40] Hamada M, Terao Y, Hanajima R, Shirota Y, Nakatani-Enomoto S, Furubayashi T, et al Bidirectional long-term motor cortical plasticity and metaplasticity induced by quadripulse transcranial magnetic stimulation. The Journal of physiology. 2008;586(16):3927–47. 10.1113/jphysiol.2008.152793 18599542 PMC2538917

[41] Fusi S, Drew PJ, Abbott LF. Cascade models of synaptically stored memories. Neuron. 2005;45(4):599–611. 15721245 10.1016/j.neuron.2005.02.001

[42] Montgomery JM, Madison DV. Discrete synaptic states define a major mechanism of synapse plasticity. Trends Neurosci. 2004;27(12):744–50. . 15541515 10.1016/j.tins.2004.10.006

[43] Emond MR, Montgomery JM, Huggins ML, Hanson JE, Mao L, Huganir RL, et al AMPA receptor subunits define properties of state-dependent synaptic plasticity. J Physiol. 2010;588(Pt 11):1929–46. 10.1113/jphysiol.2010.187229 20351044 PMC2901981

[44] Lee MC, Yasuda R, Ehlers MD. Metaplasticity at single glutamatergic synapses. Neuron. 2010;66(6):859–70. 10.1016/j.neuron.2010.05.015 20620872 PMC2911980

[45] Montgomery JM, Madison DV. State-dependent heterogeneity in synaptic depression between pyramidal cell pairs. Neuron. 2002;33(5):765–77. . 11879653 10.1016/s0896-6273(02)00606-2

[46] Vlachos A, Müller-Dahlhaus F, Rosskopp J, Lenz M, Ziemann U, Deller T. Repetitive magnetic stimulation induces functional and structural plasticity of excitatory postsynapses in mouse organotypic hippocampal slice cultures. J Neurosci. 2012;32(48):17514–23. Epub 2012/12/01. 10.1523/JNEUROSCI.0409-12.2012 . 23197741 PMC6621866

[47] Xu Z, Chen R-Q, Gu Q-H, Yan J-Z, Wang SH, Liu S-Y, et al Metaplastic Regulation of Long-Term Potentiation/Long-Term Depression Threshold by Activity-Dependent Changes of NR2A/NR2B Ratio. J Neurosci. 2009;29(27):8764–73. 10.1523/JNEUROSCI.1014-09.2009 19587283 PMC6664898

[48] Philpot BD, Cho KK, Bear MF. Obligatory Role of NR2A for Metaplasticity in Visual Cortex. Neuron. 2007;53(4):495–502. . 17296552 10.1016/j.neuron.2007.01.027 PMC1847797

[49] Quinlan EM, Philpot BD, Huganir RL, Bear MF. Rapid, experience-dependent expression of synaptic NMDA receptors in visual cortex in vivo. Nature neuroscience. 1999;2(4):352–7. . 10204542 10.1038/7263

[50] Ziemann U, Siebner H. Modifying motor learning through gating and homeostatic metaplasticity. Brain stimulation. 2008;1(1):60–6. 10.1016/j.brs.2007.08.003 20633369

[51] Quartarone A, Rizzo V, Bagnato S, Morgante F, Sant'angelo A, Romano M, et al Homeostatic-like plasticity of the primary motor hand area is impaired in focal hand dystonia. Brain. 2005;128:1943–50. . 15872016 10.1093/brain/awh527

[52] Kang J-S, Terranova C, Hilker R, Quartarone A, Ziemann U. Deficient homeostatic regulation of practice-dependent plasticity in writer’s cramp. Cereb Cortex. 2011;21(5):1203–12. 10.1093/cercor/bhq204 20974689

[53] Wankerl K, Weise D, Gentner R, Rumpf JJ, Classen J. L-type voltage-gated Ca2+ channels: a single molecular switch for long-term potentiation/long-term depression-like plasticity and activity-dependent metaplasticity in humans. J Neurosci. 2010;30(18):6197–204. 10.1523/JNEUROSCI.4673-09.2010 20445045 PMC6632730

[54] Maggio N, Vlachos A. Synaptic plasticity at the interface of health and disease: New insights on the role of endoplasmic reticulum intracellular calcium stores. Neuroscience. 2014;281C:135–46. 10.1016/j.neuroscience.2014.09.041 . 25264032

[55] Cheyne JE, Montgomery JM. Plasticity-dependent changes in metabotropic glutamate receptor expression at excitatory hippocampal synapses. Molecular and cellular neurosciences. 2008;37(3):432–9. 10.1016/j.mcn.2007.10.015 . 18191411

[56] Nitsche MA, Roth A, Kuo M-F, Fischer AK, Liebetanz D, Lang N, et al Timing-Dependent Modulation of Associative Plasticity by General Network Excitability in the Human Motor Cortex. J Neurosci. 2007;27(14):3807–12. 17409245 10.1523/JNEUROSCI.5348-06.2007 PMC6672399

